# Targeting SHP2 phosphatase in Myeloproliferative Neoplasms

**DOI:** 10.18632/oncotarget.665

**Published:** 2012-10-08

**Authors:** Raghuveer Singh Mali, Rebecca Chan, Reuben Kapur

**Affiliations:** Department of Pediatrics, Indiana University School of Medicine, Indianapolis; Department of Pediatrics, Indiana University School of Medicine, Indianapolis; Department of Pediatrics, Indiana University School of Medicine, Indianapolis

Myeloproliferative neoplasms (MPNs) are clonal hematologic disorders which result in enhanced production of immature and mature myeloid lineage-derived cells. Included in this category are the non-classical MPNs, systemic mastocytosis (SM) and juvenile myelomonocytic leukemia (JMML)[1]. Activating mutations of *KIT* (KITD816V in humans and KITD814V in mice), encoding the receptor for stem cell factor (SCF), are found in over 90% of patients with SM[2]. Activating mutations of *PTPN11*, encoding the protein tyrosine phosphatase, Shp2, are found in 35% of patients with JMML, and children bearing *PTPN11* mutations suffer a particularly poor prognosis. Somatic oncogenic *PTPN11* and *KIT* mutations are also found in myelodysplastic syndrome (MDS), acute myeloid leukemia (AML), and core binding factor-acute myeloid leukemia (CBF-AML). SM is characterized as a clonal expansion of myelomastocytic progenitors within tissues eventually resulting in organ failure and death[2], and no curative therapies are currently available. Importantly, targeting the activated version of KITD814V receptor alone has been largely ineffective; therefore, targeting downstream signaling pathways from KIT is likely to be a prudent therapeutic approach for treating SM. Likewise, JMML is a difficult-to-treat childhood MPN characterized by hematopoietic progenitor hypersensitivity to granulocyte macrophage-colony stimulating factor (GM-CSF). The only curative therapy is allogeneic hematopoietic stem cell transplantation; however, approximately half of the children will relapse after this aggressive therapeutic intervention.

While it is clear that Shp2 gain of function (GOF) mutations contribute to non-classical MPN; wildtype (WT) Shp2 is also overexpressed and hyperactive in MPNs including in chronic myeloid leukemia (CML) and more recently in cells bearing an oncogenic form of KIT (KITD816V/KITD814V) associated with SM[3]. Therefore, targeting Shp2 is likely to be of therapeutic benefit not only for treating JMML but also for diseases bearing an oncogenic form of KIT including SM. However, targeting Shp2 with small molecule inhibitors has been challenging, in part due to conservation of the active site pocket, which is shared between several other members of the tyrosine phosphatase family. Recent studies have described the characterization and functional efficacy of some Shp2 inhibitors. To this end, NSC-87877 was described as a Shp2 inhibitor; however, it also demonstrates activity towards the related protein tyrosine phosphatase, SHP1[4]. PHPS1 demonstrates specificity for Shp2 over Shp1[5]. Additional small molecule inhibitors of Shp2 have also been described that show in vitro functional efficacy, although at very high concentrations[6]. More recently, Zhang et al described a salicyclic acid based Shp2 inhibitor, IIB08[7]. IIB08 binds both the active site of Shp2 as well as the adjacent spanning sub-pockets and demonstrates enhanced overall affinity and selectivity for Shp2. Early in vitro studies showed that IIB08 inhibits GM-CSF induced growth of bone marrow cells bearing GOF Shp2 mutations (D61Y and E76K) commonly found in patients with JMML[7]. More recently, Mali et al extended these findings to demonstrate that IIB08 treatment of cells bearing an oncogenic form of KIT (KITD814V) results in reduced Shp2 constitutive phosphorylation, repression in ligand independent growth and survival[3]. Importantly, they also demonstrated that complete deficiency of Shp2 in bone marrow cells bearing the oncogenic KIT phenocopies the observed growth repression seen in the presence of IIB08. Biochemically, the authors showed that constitutive binding of Shp2 to p85α regulatory subunit of class IA PI3Kinase and Gab2 in KITD814V expressing cells is significantly disrupted in the presence of IIB08 resulting in reduced activation of AKT and ERK MAPK. In vivo, mice bearing KITD814V expressing cells had enhanced survival when treated with IIB08, which was further prolonged when these mice were treated with a combination of IIB08 and a PI3Kinase inhibitor, suggesting that targeting Shp2 in vivo in combination with a PI3Kinase inhibitor is likely to be of therapeutic benefit for some MPNs[3].

While identifying Shp2 as a potential therapeutic target for treating KITD814V bearing MPNs is exciting; identifying the appropriate cellular populations that need targeting in SM is equally crucial. SM is a heterogeneous disease. In addition to assuming an indolent course, SM can also take an aggressive direction (as in aggressive systemic mastocytosis or mast cell leukemia) or can be associated with a non-mast cell hematologic malignancy (associated clonal hematological non-mast cell lineage disease, SMAHNMD)[2]. Although at present it is unclear how a single mutation results in such diverse cellular outcomes; somatic oncogenic KIT mutations associated with mastocytosis have been identified in relatively primitive stem and progenitor cells as well as in more mature mast cells. Given the fact that Shp2 is expressed in both primitive hematopoietic progenitors and committed mast cells[3, 8], genetic studies involving the deletion of Shp2 in relevant cell types to identify whether Shp2 plays quantitatively or qualitatively distinct role in regulating oncogenic KIT-induced disease heterogeneity will be essential. To this end, Gerbaulet et al[9] have recently described a mouse model driven by an inducible expression of KITD814V, which mimics several cardinal features of human mastocytosis including cutaneous mastocytosis (CM) as well as SM. This model permits the examination and pathologic consequences of KITD814V expression in immature hematopoietic progenitors (relevant for SM) vs. that of committed mast cells (relevant for cutaneous mastocytosis). Utilizing this model and conditionally deleting Shp2 in stem cells vs. mast cells should provide a clearer picture with respect to the precise role Shp2 plays in KITD814V induced disease heterogeneity (Figure 1).

**Figure:**
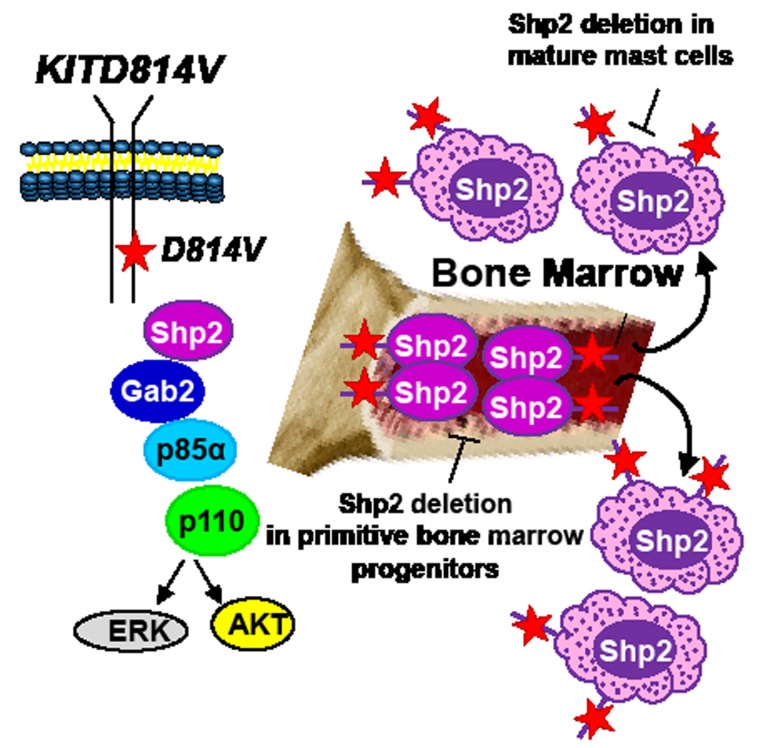


In addition to identifying the appropriate cellular population(s) for targeting Shp2 in MPNs, it will be essential to determine if hyperactive WT Shp2 similarly contributes to MPN as GOF Shp2. This is an important issue, since the structural requirements and substrate specificity of GOF Shp2 could be different from that of a hyperactive form of WT Shp2 and, thus, targeting Shp2 in different MPNs may require different strategies. Shp2 comprises of several functional domains, including N- and C-SH2 domains, a PTP domain and C-terminal tyrosine (542 and 580) residues. While the phosphatase function of Shp2 has been shown to be important for Ras activation, germline PTPN11 mutations causing loss of phosphatase activity yield similar phenotypic anomalies to that induced by germline GOF PTPN11 mutations, exemplified by individuals with LEOPARD syndrome and Noonan syndrome, respectively[10]. Consistently, structure-function studies performed in Shp2-deficient zebrafish embryos demonstrate that Shp2 possesses both phosphatase-dependent and -independent roles in regulating neural crest specification, migration, and apoptosis. Furthermore, inhibition of ERK activity did not result in cell death, suggesting that Shp2-induced ERK activation may not be essential for survival. It is quite possible that Shp2 molecules bearing a GOF mutation, rendering a constitutively open conformation, results in both quantitative as well as qualitative differences in the utilization of its SH2 domains, PTP domain, and C-terminal tyrosine residues (Y542 and Y580) compared to WT Shp2 activated in response to oncogenic KIT. It is important to identify the individual role(s) of these domains in transformation, as targeting specific domains of Shp2 as opposed to inhibiting only the phosphatase function is likely to be a more prudent approach for treating diseases involving abnormal Shp2. Taken together, a combined understanding of Shp2's structural requirement as well as defining the appropriate cellular population(s) for targeting Shp2 is likely to aid in the design of better Shp2 inhibitors in the future.
